# Direct physical interaction of active Ras with mSIN1 regulates mTORC2 signaling

**DOI:** 10.1186/s12885-019-6422-6

**Published:** 2019-12-19

**Authors:** Mehraj-U-Din Lone, Javed Miyan, Mohammad Asif, Showkat A. Malik, Parul Dubey, Varsha Singh, Kavita Singh, Kalyan Mitra, Deepali Pandey, Wahajul Haq, Himanshi Amita, Prince Kumar Singh, Wieland Kiess, Franziska Kaessner, Antje Garten, Smrati Bhadauria

**Affiliations:** 1 Division of Toxicology and Experimental Medicine, Central Drug Research Institute (CSIR), Lucknow, Uttar Pradesh 226031 India; 2 Academy of Scientific and Innovative Research (AcSIR), New Delhi, 110025 India; 3 Department of Surgical Oncology, King George Medical University, Lucknow, Uttar Pradesh 226003 India; 4 Electron Microscopy Unit, Sophisticated Analytical Instrumentation Facility, Central Drug Research Institute (CSIR), Lucknow, Uttar Pradesh 226031 India; 5 Medicinal and Process Chemistry Division, Central Drug Research Institute (CSIR), Lucknow, Uttar Pradesh 226031 India; 6 Center for Pediatric Research Leipzig, University Hospital for Children and Adolescents, Faculty of Medicine, University of Leipzig, Leipzig, Germany; 7 Institute of Metabolism and Systems Research, University of Birmingham, Birmingham, UK

**Keywords:** Cancer, Mammalian target of rapamycin (mTOR), Signaling, Ras, Superoxide anion

## Abstract

**Background:**

The mechanistic (or mammalian) target of rapamycin (mTOR), a Ser/Thr kinase, associates with different subunits forming two functionally distinct complexes, mTORC1 and mTORC2, regulating a diverse set of cellular functions in response to growth factors, cellular energy levels, and nutrients. The mechanisms regulating mTORC1 activity are well characterized; regulation of mTORC2 activity, however, remains obscure. While studies conducted in *Dictyostelium* suggest a possible role of Ras protein as a potential upstream regulator of mTORC2, definitive studies delineating the underlying molecular mechanisms, particularly in mammalian cells, are still lacking.

**Methods:**

Protein levels were measured by Western blotting and kinase activity of mTORC2 was analyzed by in vitro kinase assay. In situ Proximity ligation assay (PLA) and co-immunoprecipitation assay was performed to detect protein-protein interaction. Protein localization was investigated by immunofluorescence and subcellular fractionation while cellular function of mTORC2 was assessed by assaying extent of cell migration and invasion.

**Results:**

Here, we present experimental evidence in support of the role of Ras activation as an upstream regulatory switch governing mTORC2 signaling in mammalian cancer cells. We report that active Ras through its interaction with mSIN1 accounts for mTORC2 activation, while disruption of this interaction by genetic means or via peptide-based competitive hindrance, impedes mTORC2 signaling.

**Conclusions:**

Our study defines the regulatory role played by Ras during mTORC2 signaling in mammalian cells and highlights the importance of Ras-mSIN1 interaction in the assembly of functionally intact mTORC2.

## Background

The mammalian target of rapamycin (mTOR), an evolutionary conserved Ser/Thr kinase belonging to phosphatidylinositol 3-kinase-related kinase (PIKK) family, regulates a myriad of anabolic and catabolic cellular processes by forming two functionally and structurally distinct complexes, i.e. mTORC1 and mTORC2 [1]. Both the mTOR complexes contain shared as well as unique components. The mTOR, mammalian lethal with SEC13 protein 8 (mLST8) and DEP domain-containing mTOR interacting protein (DEPTOR) are commonly present in both the complexes. The regulatory-associated protein of mTOR (Raptor), proline-rich AKT substrate 40 (PRAS40) are unique to mTORC1 while rapamycin-insensitive companion of mTOR (Rictor), mammalian stress-activated protein kinase (SAPK)-interacting protein 1 (mSIN1) and protein observed with Rictor (PROTOR) are exclusive to mTORC2 [2]. Rheb (Ras homolog enriched in brain) and Rag, small GTP-binding proteins, primarily regulate mTORC1 [3, 4], however, the mechanism of mTORC2 activation is not known.

Rapamycin interacts with and inhibits mTORC1 [5]. In contrast, mTORC2 is insensitive to acute rapamycin treatment [6]. The mTOR signaling is deregulated in most cancer types. Consequently, mTOR is being actively pursued as a potential anti-cancer target leading to the emergence of rapamycin and rapalogs as potential anti-cancer agents. Though clinical trials using rapalogs demonstrated clinical benefits in several cancer types, the objective response rates achieved with single-agent therapy have been only modest [7]. Furthermore, for tumors with prevalent PI3K/AKT activating mutations, such as glioblastoma, prostate and breast cancers, significant improvement in response to rapamycin/rapalogs is rarely observed. In fact, a large body of data indicates that rapamycin exerts only cytostatic effects and often its use culminates into refractory/resistant tumors [8, 9]. The ineffectiveness of rapamycin as single-agent therapy is partly attributable to mTORC1-dependent negative feedback loops that are inactivated following mTORC1 inhibition. Loss of these feedback inhibition loops overrides the partial inhibitory (only mTORC1 and not mTORC2) effect and promotes survival [7, 10]. The mTORC2 phosphorylates AKT at Ser^473^ resulting in a tenfold increase in its catalytic activity [11]. The mTORC2-dependent AKT phosphorylation at Ser^473^ and resultant AKT activation compromises the inhibitory effects of rapamycin and promotes survival [12]. The mTORC2 dependent AKT Thr^450^ phosphorylation is a key determinant for controlling the cellular turnover of AKT protein. Heightened mTORC2 activation (during rapamycin therapy) may results in Thr^450^ phosphorylation-mediated stabilization of AKT, resulting in further attenuation of therapeutic effectiveness [11, 13].

Therefore, in order for anti-mTOR therapy to be efficacious, simultaneous and/or prior inhibition of mTORC2 is well warranted. In fact, several recent studies propose that selective and specific targeting of mTORC2 could be a better anti-cancer strategy than mTORC1 inhibition because such inhibitors would not perturb the mTORC1-dependent negative feedback loops and thus could have a more acceptable therapeutic window [7]. However, lack of knowledge about upstream regulation limits the possibility of selectively targeting mTORC2. Although several reports suggest the role of Ras protein in mTORC2 regulation [14], the definitive experimental evidence is yet to be presented.

This study aims to decipher the upstream regulatory mechanisms for mTORC2 assembly and function. We recently reported that ER-mediated functional alteration of MnSOD and ensuing build-up of superoxide anion (O_2_^.-^) resulted in mTORC2 activation in breast cancer cells [15, 16]. Pertinent to this, superoxide anions have been reported to activate Ras through a radical-based mechanism similar to that of nitric oxide [17]. One of the structural components of functionally intact mTORC2, viz. mSIN1 binds to active H-Ras through a region corresponding to amino acids 260–460 and inhibits Ras signaling [18]. The corresponding interacting domain on Ras has also been identified as a putative RAF-like Ras-binding domain (RBD) [19]. In view of this, we set out to study the role of Ras as an upstream regulatory element of mTORC2. The present study could pave the way for a better understanding of mTORC2 signaling and provide the basis for the designing mTORC2 specific inhibitors which in turn could be used as an effective combinatorial therapeutic regimen against cancer.

## Methods

### Antibodies and reagents

Rabbit anti-p-Ser^2481^mTOR monoclonal, Rabbit anti-mTOR monoclonal, Rabbit anti- p-Ser^473^AKT monoclonal, Rabbit anti-AKT monoclonal, Rabbit anti-PKC-α monoclonal, and Rabbit anti-Rictor monoclonal were purchased from Cell Signaling Technology (Beverly, MA; USA). Goat anti-p-Ser^657^PKC-α polyclonl, mouse anti-β-actin monoclonal, mouse anti-HA monoclonal and rabbit anti-SOD2 polyclonal were purchased from Santa Cruz Biotechnology (Santa Cruz, CA, USA). Rabbit anti-Ras monoclonal, Mouse anti-Transferrin monoclonal, Alexa Fluor-488 conjugated goat-anti-mouse, and Alexa Fluor-555 conjugated goat-anti-rabbit, were purchased from Thermo Fisher Scientific (Waltham, MA, USA). Rapamycin and mouse anti- mSIN1 were purchased from Millipore (Billerica, MA, USA). HRP-conjugated goat anti-rabbit/anti-mouse was procured from Jackson Immuno Research Europe Ltd. (UK). PLA kit was procured from Sigma (USA). Aldrich. Pyrogallol was procured from SD Fine Chemicals (Mumbai, India). Mn (III)tetrakis (4-benzoic acid) porphyrin chloride (MnTBAP) was procured from Calbiochem (Billerica, MA, USA). GTP-γ-S and Farnesyltransferase inhibitor (FTI) (Lonafarnib) were procured from Abcam (Cambridge, UK). Geranylgeranyltransferase inhibitors (GGTI) were procured from Sigma-Aldrich (St. Louis, Missouri, USA). Protein A/G sepharose beads were purchased from BioVision Inc. (Cara ct, PA, USA). Matrigel was procured from BD Biosciences.

### Cell culture and treatment

MDA-MB-231(ATCC^(R)^HTB-26), DU 145 (ATCC^(R)^HTB-81), PC-3 (ATCC^(R)^CRL-1435), and MCF7 (ATCC^(R)^HTB-22) cells were obtained from ATCC (Manassas, VA, USA). Cell lines were authenticated by through STR profiling for total ten Genetic loci followed by querying against reference genotypes available in ATCC® and DSMZ® reference cell line STR databases. Mycoplasma contamination was tested at institutional cell repository facility. MDA-MB-231 and DU 145 cells were cultured in RPMI 1640 media supplemented with 100 μg/ml penicillin, 100 μg/ml streptomycin and 10% FBS. PC-3 and MCF7 cells were grown in Dulbecco’s modified Eagle’s medium (DMEM) containing 10% FBS and antibiotic. All cells were maintained in a humidified atmosphere (95% humidity) at 37 °C and 5% CO_2_.

### Lipoma and preadipocyte cell culture

Lipoma cell cultures (LipPD1–3) used in this study were obtained from lipomatous adipose tissue that was resected for diagnostic and therapeutic reasons. Control primary pre-adipocytes were removed from adipose tissue obtained from pediatric or young adult patients during routine surgery. Written informed consent was provided by the parents of all patients enrolled in the present study. All patients were admitted to the Hospital for Child and Adolescent Medicine (Leipzig University, Leipzig, Germany) and samples were collected between July 2007 and December 2016. Patients with morbidities other than PHTS were excluded.

All cell strains were established according to protocols as described previously [20, 21]. Cells were maintained in DMEM/F12 medium supplemented with 10% fetal calf serum, glutamine (2 mM), biotin (33 mM) and pantothenic acid (17 mM; all from Biochrom, Ltd., Cambridge, UK) at 37 °C in a humidified atmosphere containing 5% CO_2_.

### Plasmids and siRNA

The pcDNA-3SOD2 was a kind gift from Dr. Alfred S Lewin (University of Florida, Gainesville, FL, USA). Wild-type pcDNA3.1-C-(k)DYK-SIN1 and mutant pcDNA3.1-C-(k)DYK-SIN1 were synthesized by GenScript Biotech Corp. (Piscataway, NJ). The pcDNA3-HA-H-Ras_wt and paGFP-HRas-(G12 V) were procured from Addgene (Cambridge, MA). The siRNA experiments were carried out using a set of pre-validated siRNAs that were directed against SOD2 and Rictor (Eurofins Analytik, Germany). All transfections were carried out using Lipofectamine 3000 reagent from Thermo Fisher Scientific (USA).

### Subcellular fractionation

Subcellular fractionation was performed in accordance with the method developed by R. Patten at Abcam (2013). Briefly, cells were lysed in subcellular fractionation buffer (250 mM Sucrose, 20 mM HEPES pH 7.4, 10 mM KCl, 1.5 mM MgCl_2_, 1 mM EDTA; 1 mM EGTA; 1 mM DTT and Protease inhibitor cocktail-III) followed by passing the sample through 25 gauge needle about 10–12 times. The sample was further incubated for 20 min. on ice so that cells were completely lysed. The cell lysate was centrifuged at 3000 rpm for 5 min. at 4 °C, and supernatant was collected and centrifuged again at 8000 rpm for 10 min at 4 °C. The resultant supernatant which contained both cytosolic and membrane fractions was subjected to ultracentrifugation at 40,000 rpm (100,000×g) for 1 h at 4 °C. The supernatant containing cytosolic fraction was collected in a fresh tube and further concentrated in a rotational vacuum concentrator for 2.5 h at 32 °C, followed by Western blot analysis. The pellet containing membrane fraction was washed twice with fractionation buffer and resuspended in 400 μl of fractionation buffer by repeated (10–12 times) passing through 25 gauge needle. The sample was again centrifuged at 40,000 rpm (100,000×g) for 45 min. at 4 °C. The pellet containing enriched membrane fraction was resuspended in 40 μl of RIPA buffer and subjected to Western blot analysis for detecting the levels of mSIN1, mTOR, and Ras.

### Western blotting

The expression level of various proteins was evaluated using Western blotting. Briefly, cells were lysed in cold radioimmunoprecipitation assay (RIPA) buffer containing protease and phosphatase inhibitors. Cell lysates were incubated on ice for 10 min, sonicated on ice for 30 s, and centrifuged at 12,000 rpm for 15 min at 4 °C. After protein isolation, estimation was done by Lowry’s method. Proteins in cell lysates (or immunoprecipitated samples) were resolved through SDS-PAGE and were transferred to PVDF membranes. After blocking with 5% BSA in TBST, membranes were incubated at 4 °C overnight with specific antibodies. The next day, blots were incubated with a horseradish peroxidase-conjugated secondary antibody. Blots were developed with the ECL chemiluminescence substrate and visualized on Image Quant 4000.

### Co-immunoprecipitation and recombinant protein pull-down assay

Whole-cell extracts were prepared in non-denaturing CHAPS immunoprecipitation buffer (40 mM HEPES of pH 7.4, 120 mM NaCl, 2 mM EDTA, 0.3% CHAPS, 10 mM pyrophosphate, 10 mM glycerophosphate, 50 mM NaF) containing phosphatase and protease inhibitors. Sample equivalent to 200 μg of protein was incubated with Mouse anti-mSIN1 monoclonal antibody and Rabbit anti-Ras monoclonal antibody overnight at 4 °C. The samples were then incubated with 30 μl of protein sepharose A/G beads for another 4 h at 4 °C. Immunoprecipitates were washed five times with CHAPS immunoprecipitation buffer and elution was carried out with 2X sample buffer. Haemagglutinin HA-tagged *RAS* and Flag-tagged *mSIN1* pull-down assays were carried out using standard manufactures protocol (Clontech/Thermo Scientific).

### In situ proximity ligation assay

The assay was performed on paraformaldehyde-fixed MDA-MB-231 cells employing Duolink PLA555 Kit (Olink Biosciences, Uppsala, Sweden) in accordance with manufacturer’s protocol. Briefly, the control and treated MDA-MB-231 cells seeded on glass coverslips were fixed using PBS-paraformaldehyde (4%) followed by permeabilization with PBS-TritonX-100 (0.5%) and blocked with blocking solution (provided with the kit) for 30 min in a preheated humidity chamber at 37 °C. Thereafter cells were incubated in a humidity chamber overnight at 4 °C with anti-human mSIN1 mouse IgG and anti-human Ras rabbit IgG antibody (1:100). Cells were then incubated with oligonucleotide-labeled anti-mouse and anti-rabbit secondary antibodies (PLA probes) for 1 h in a pre-warmed humidity chamber at 37 °C. Cover-slips harboring cells were washed two times with wash buffer A (provided with the kit) and thereafter incubated with 50 μl of the ligation-ligase mixture in a preheated humidity chamber at 37 °C for 30 min. Following this, the ligation-ligase solution was removed and a 40 μl amplification buffer was added. Samples were incubated for 100 min in a preheated humidity chamber at 37 °C. Finally, after washing two times with wash buffer B (provided with the kit), sections were mounted in aqueous mounting media containing DAPI. Cells were visualized in Olympus BX61-FV1200-MPE (Tokyo, Japan), and images were analyzed with Image J software.

### Cell lysis and immunoprecipitation-based isolation of mTORC2 and AKT

The in vitro mTORC2 kinase assay was carried out using mTORC2 isolated from control and 20 μM Pyrogallol-treated MDA-MB-231 cells lysates. Isolation was done according to Zhou and Huang (2011) with slight modifications [22]. Briefly, the cells were harvested and lysed in non-denaturing CHAPS lysis buffer (40 mM HEPES of pH 7.4, 120 mM NaCl, 2 mM EDTA, 0.3% CHAPS, 10 mM pyrophosphate, 10 mM glycerophosphate, 50 mM NaF) containing protease and phosphatase inhibitor cocktails [23]. The lysates were centrifuged at 16,500 g for 10 min at 4 °C and supernatant was utilized for isolation of mTORC2 using Rabbit anti-Rictor monoclonal antibody directed co-immunoprecipitation. Subsequently, protein G beads containing mTORC2 immunocomplexes were washed four times with CHAPS lysis buffer at 6000 g for 1 min at 4 °C, followed by a single wash with mTORC2 kinase buffer containing 25 mM HEPES of pH 7.4, 100 mM potassium acetate, and 1 mM MnCl_2_ [23], and utilized for in vitro kinase assay.

Isolation of AKT (to serve as substrate) was also carried out through anti-AKT rabbit monoclonal antibody directed immunoprecipitation from a whole-cell lysate of MDA-MB-231 cells that were switched to CS-FBS containing media for 24 h prior to harvest. Furthermore, to ensure that immunoprecipitated AKT remained dephosphorylated at Ser^473^ to serve as a substrate during the kinase reaction, 2 h prior to harvesting, the cells were treated with Wortmannin (10 μM), a potent PI3K selective inhibitor.

### Kinase assay and Immunoblot analysis

The mTORC2 and AKT immunocomplexes isolated as described above were utilized for in vitro kinase assay [24, 25]. For in vitro kinase assay, AKT was eluted from protein G beads and ~ 6.5 μg protein equivalent of eluent was equilibrated in mTORC2 kinase buffer for 5 min on ice in presence of protein G immunocomplexes of mTORC2 (~ 40 ng). A parallel reaction mixture containing all the components except for protein G beads immunocomplexes of mTORC2 was considered as blank. The reaction was initiated by addition of ATP (100 μM) and transferring the tubes at 30 °C in shaking water bath at 100 rpm for 2 h. The reaction was terminated by adding 10 mM EDTA for 5 min at room temperature. Subsequently, the reaction mixture was denatured by adding an equal volume of sample buffer followed by boiling for 5 min. Thereafter, aliquots of the reaction mixture were resolved through 6% (for mTOR) and 10% (for AKT) denaturing polyacrylamide gel, followed by Western blotting for detecting the levels of p-Ser^473^AKT, total-AKT, p-Ser^2481^mTOR, and total-mTOR.

### Peptide synthesis and purification

The rationale based peptide synthesis (Additional file 1: Figure S3) was carried out using a sequence of RBD on mSIN1 as reported by Schroeder et al. [19]. These moderately lipophilic peptides comprising generic amino acids often show cell membrane permeability unless in specific cases, where fail to cross the cell membrane. The synthesis was carried out by standard Fmoc based solid-phase peptide synthesis (SPPS) on Rink amide resin. The crude peptides were purified by reversed-phase HPLC (RP-HPLC) using a C-18 column and then the samples were lyophilized. Pure peptides were analyzed by analytical RP-HPLC and characterized by mass and NMR spectrometry.

### Immunofluorescence and confocal microscopy

Cells were seeded on glass coverslips and cultured in RPMI media with 10% FBS. They were incubated in media containing 10% CSFBS for another 24 h before the treatment. Cells were then fixed with PBS-paraformaldehyde (4%), permeabilized with PBS-TritonX-100 (0.5%), blocked for 1 h with 3% BSA and incubated with respective primary antibodies overnight at 4 °C. The next day, cells were incubated with corresponding Alexafluor 555 conjugated secondary antibody, while DAPI was used to stain the nucleus. Subsequently, cells were visualized under oil immersion using a Carl Zeiss LSM 510 META (Carl Zeiss, Oberkochen, Germany). Images were analyzed using the Zeiss LSM Data Server software.

### Cell migration assay

5.0 × 10^4^ cells were seeded in a 24-well cell culture plate. At 30% confluency, the culture media was switched to that containing 10% CS-FBS. After 24 h scrambled Peptide and P4 treatment given for further 24 h. After 24 h, cell monolayers of each well were scratched with a sterile 200 μl pipette tip. The respective media was harvested, centrifuged to sediment non-adherent/scrapped out cells. The cell pellet was discarded, while clear supernatant was carefully collected, and replenished back into corresponding wells. Simultaneously, Pyrogallol (20 μM) was added to designated wells, and cells were further incubated for 12 and 24 h at 37 °C under standard cell culture conditions. Thereafter, cells were visualized under phase contrast Leica DFC450 C inverted microscope.

### Cell invasion assay

For assessing the effect of P4 on the invasive potential of MDA-MB-231 cells, matrigel transwell invasion assay was carried out. 2 × 10^4^ cells were seeded onto Matrigel-coated upper chamber of transwell cell culture inserts (1.0 μM PET, Millipore), and introduced into standard 12-well cell culture plates. The lower chamber was filled with complete media. The cells were incubated with P4 (24 h prior treatment) and Pyrogallol either alone or in combination at 37 °C for designated time periods so as to allow them to migrate through the Matrigel-coated PET membrane. Thereafter, inserts harboring cancer cells were collected, the non-invaded cells at the top side were scraped off using cotton swabs. The invading cells i.e. the ones adhered to the underside of the membrane were fixed in 4% paraformaldehyde, mounted in aqueous mounting media containing DAPI and visualized under Leica DFC450 C fluorescence microscope. Cells were counted using Image J software. The quantification was carried out in five different fields of three replica sets.

### Statistical analysis

Data were summarized as Mean and SEM, and the graphs were generated using GraphPad Prism 6.0 Quantification (relative density of blots) was performed by densitometry using myimage Analysis software (Thermo Scientific). One-way analysis of variance (ANOVA) followed by Newman–Keuls post hoc test were employed for comparisions. A two-tailed *p* ≤ 0.05 was considered statistically significant.

## Results

### Superoxide anion upregulation accounts for heightened mTORC2 signaling

**Fig. 1:**
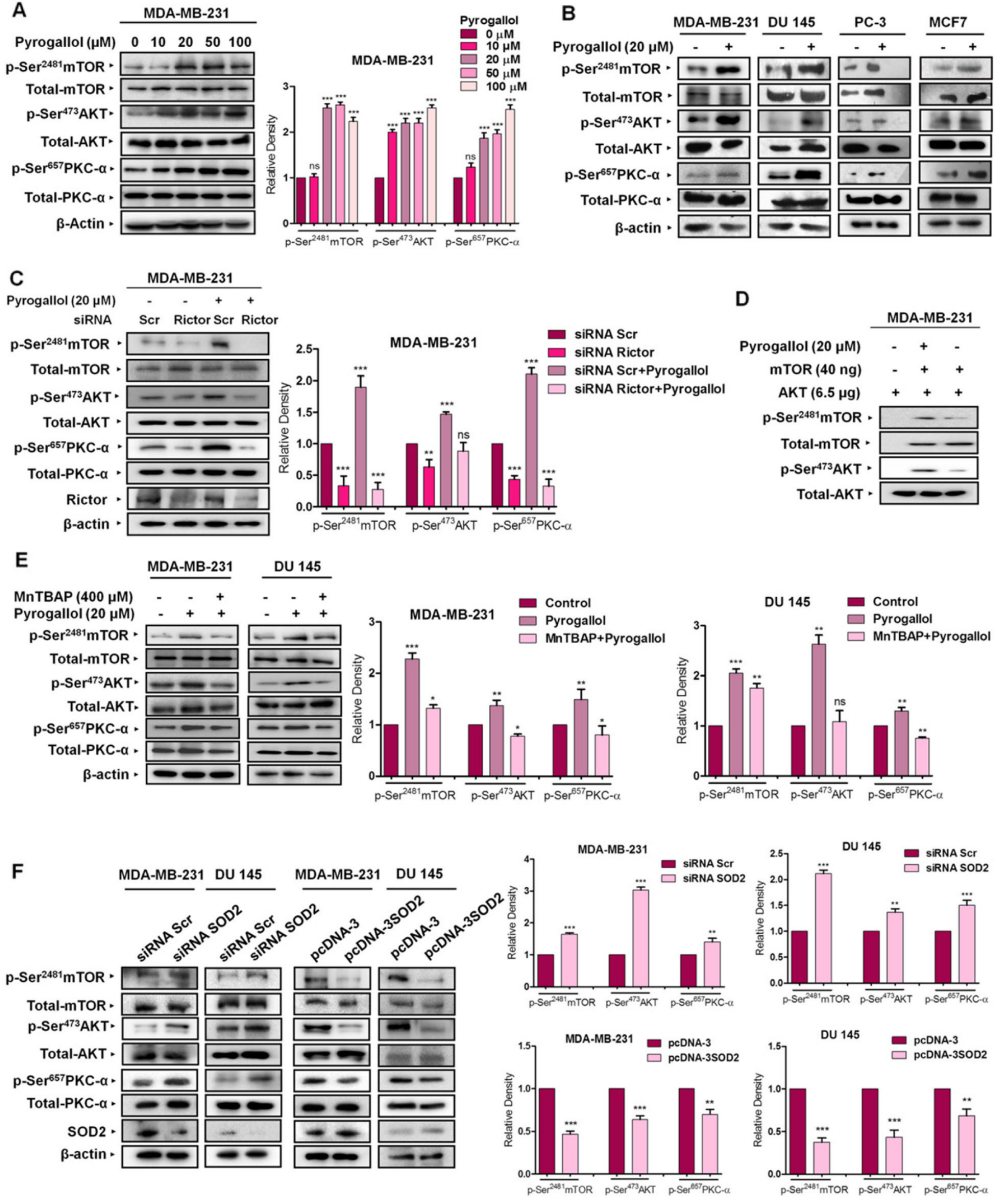
Superoxide anions potentiate mTORC2 signaling. **a** Pyrogallol treated MDA-MB-231 cells exhibited elevated mTORC2-specific markers, pSer^2481^-mTOR, pSer^473^-AKT, and pSer^657^-PKC-α. **b** Four different cell lines viz. MDA-MB-231, DU 145, PC-3, and MCF7 exhibited increased mTORC2 signaling following Pyrogallol (20 μM) treatment for 24 h. **c** Attenuation of mTORC2 cascade via Rictor directed siRNA resulted in diminished pSer^2481^-mTOR, pSer^473^-AKT, and pSer^657^-PKC-α despite Pyrogallol (20 μM) treatment. **d** The in vitro kinase assay showing mTORC2 immunopurified from Pyrogallol treated MDA-MB-231 cells exhibited elevated kinase activity as compared to mTORC2 purified from non-treated cells. **e** Pre-treatment with mitochondria permeating superoxide anion quencher MnTBAP attenuated Pyrogallol (20 μM) stimulated mTORC2 signaling. **f** Modulating superoxide anion levels indirectly by gene-based downregulation/upregulation of mitochondrial resident SOD2 resulted in potentiated/attenuated mTORC2 signaling respectively. All data are representative of three independent experiments. ns, not significant. *P ≤ 0.05, **P ≤ 0.01 and ***P ≤ 0.001

Our previous study conducted on breast cancer cells established superoxide anions as a key mediator of mTORC2 activation following 17-β-estradiol treatment [15]. Accordingly, in the current study, we first set out to substantiate if superoxide anion per se may activate the mTORC2 pathway. Pyrogallol, a polyphenol compound, and a potent superoxide anion generator is widely employed as an agent for assessing the effect of superoxide anions [26]. Therefore, Pyrogallol was utilized for investigating the effect of superoxide anions with respect to mTORC2 signaling. We first set out to deduce the optimum concentration of Pyrogallol, at which cells would likely exhibit elevated O_2_^.-^ levels without any considerable loss of vitality. Accordingly, MDA-MB-231 cells were grown overnight and then switched to cell culture media containing charcoal-stripped fetal bovine serum (CS-FBS) for another 24 h followed by treatment with increasing concentrations of Pyrogallol (10, 20, 50 and 100 μM) for 24 h. We observed considerable superoxide generation at 20 μM and higher concentrations. However, at the concentration of 20 μM, cells did not exhibit any considerable loss of viability (Additional file 1: Figure S1A). Furthermore, cells exhibited considerable mTORC2 activation at this concentration (Fig. 1a). Taking a cue from this, the 20 μM concentration was selected for assessing mTORC2 activation status. With respect to the duration of the treatment, MDA-MB-231 cells were treated with 20 μM Pyrogallol for varying time periods (5 m, 15 m, 30 m, 60 m, 3 h, 6 h, 12 h, and 24 h) followed by Western blot analysis of mTORC2 specific signaling intermediates (p-Ser^2481^mTOR, p-Ser^473^AKT, and p-Ser^657^PKC-α). There was a gradual increase in the levels of functionally intact mTORC2, starting from 30 min and up until 24 h following Pyrogallol treatment (Additional file 1: Figure S1B). Taking a cue from above, the 20 μM concentration of Pyrogallol for 24 h was selected for confirming the effect across four different cell lines, namely MDA-MB-231, DU 145, PC-3 and MCF7. Pyrogallol treatment accounted for heightened mTORC2 signaling in all the four cell lines with the most prominent effect observed in MDA-MB-231 and DU 145 cell lines. Pyrogallol-treated cells exhibited elevated phosphorylation of mTOR at Ser^2481^, a marker of intact and active mTORC2 [27], as well as both PI3K-dependent and independent downstream targets, viz. AKT and PKC-α at Ser^473^ and Ser^657^ respectively (Fig. 1b;Additional file 1: Figure S5). Pyrogallol treatment failed to elicit a similar effect in cells where mTORC2 signaling was abrogated via siRNA mediated Rictor silencing. Diminished levels of p-Ser^473^AKT and p-Ser^657^ PKC-α levels clearly substantiated that Pyrogallol treatment accounted for AKT and PKC-α activation in a mTORC2 dependent manner (Fig. 1c). Similarly, in vitro kinase activity of mTORC2 immunoprecipitated from Pyrogallol treated cells was much heightened as revealed by increased phosphorylation of AKT at Ser^473^ in vitro compared to that by mTORC2 immunoprecipitated from untreated cells (Fig. 1d). Results indicate that Pyrogallol treatment accounts for increased levels of functionally intact mTORC2 compared to that in untreated cells. At the same time, Pyrogallol-induced mTORC2 activation was markedly abrogated in MDA-MB-231 and DU 145 cells that were pre-treated with MnTBAP, a superoxide dismutase 2 (SOD2) mimetic and a superoxide anion scavenger, thereby establishing the role of superoxide anion as an activator of the mTORC2 pathway (Fig. 1e).

To further confirm the involvement of superoxide anions, siRNA-mediated downregulation of cellular SOD2 levels was carried out so as to potentiate cellular levels of superoxide anions, and the effect on mTORC2 was assessed. SOD2 silencing resulted in potentiated p-Ser^2481^mTOR, pSer^473^AKT and pSer^657^PKC-α levels in MDA-MB-231 and DU 145 cell lines as compared to that in controls. Under similar conditions, ectopic overexpression of SOD2 resulted in attenuated mTORC2 signaling as evident from diminished p-Ser^2481^mTOR, p-Ser^473^AKT and p-Ser^657^PKC-α levels (Fig. 1f).

### Superoxide anion stimulated mTORC2 activation is a Ras-mediated phenomenon

**Fig. 2:**
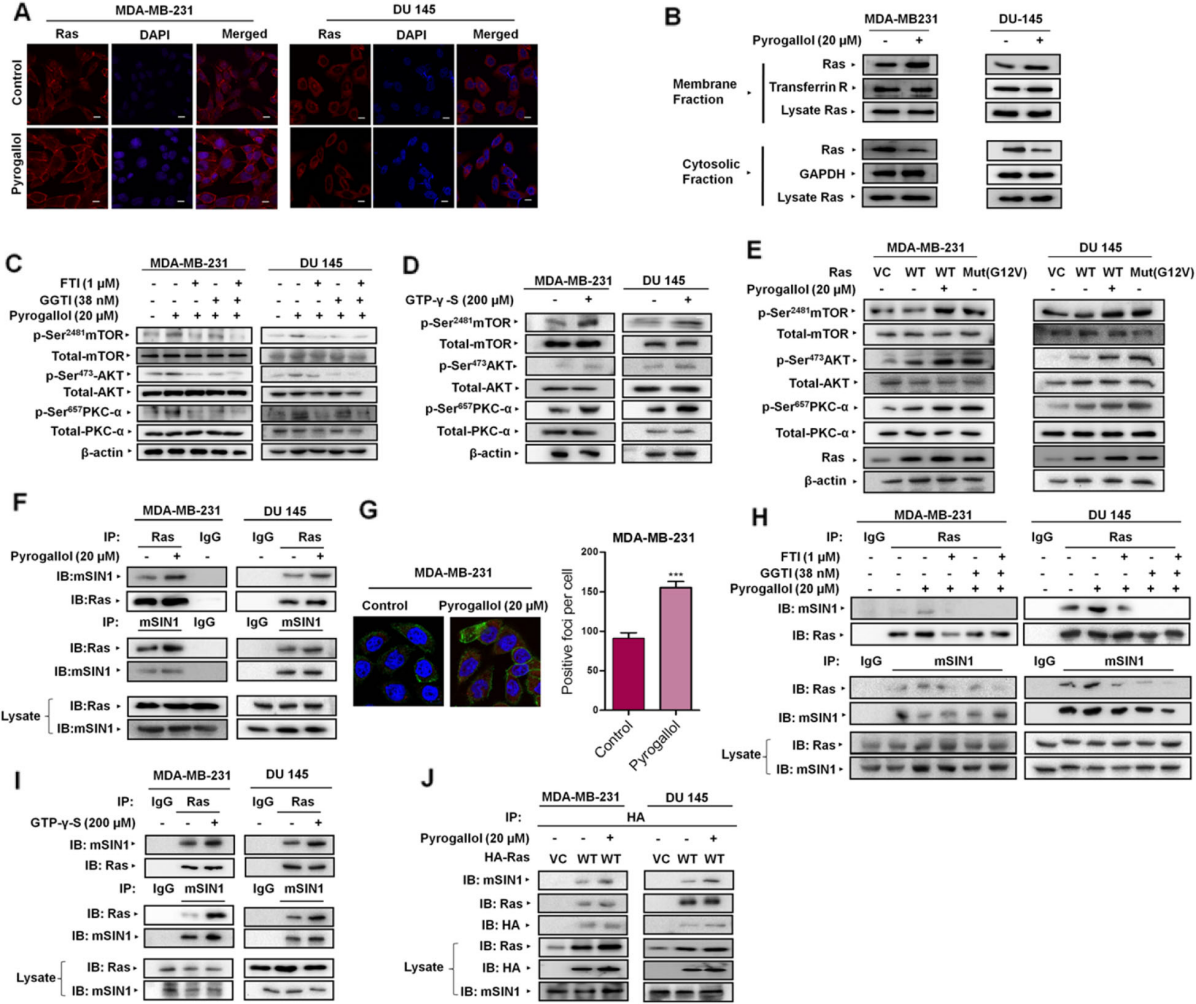
Superoxide anion stimulated the activation of mTORC2 is a Ras-mediated phenomenon. **a** Immunofluorescence micrograph depicting alteration in the cellular distribution of Ras 24 h following Pyrogallol (20 μM) treatment. Scale bars, 10 μm. **b** Subcellular fractionation followed by Western blot analysis depicting the increased distribution of Ras from cytosolic fraction to membrane fraction 24 h post-pyrogallol (20 μM) treatment. **c** MDA-MB-231 and DU 145 cells treated with Ras inhibitors FTI (1 μM) and GGTI (38 nM) for 24 h prior to Pyrogallol treatment of further 24 h exhibited impaired mTORC2 signaling. **d** An in vitro reconstitution assay wherein constitutive Ras activation was achieved by incubating MDA-MB-231 and DU145 cell lysates with GTP-γ-S (200 μM) for 12 mins revealed heightened mTORC2 signaling in the presence of active Ras. **e** MDA-MB-231 and DU 145 cells expressing wild-type H-Ras exhibited heightened mTORC2 signaling following Pyrogallol (20 μM) treatment. Comparable mTORC2 activation was observed in MDA-MB-231 and DU 145 cells, expressing constitutively active, mutant H-Ras (G12 V). **f** Co-immunoprecipitation followed by immunoblot analysis showing an increased association of Ras with mSIN1 in MDA-MB-231 and DU145 cells treated with Pyrogallol (20 μM) for 24 h. **g** In situ proximity, ligation assay revealed direct physical interaction between Ras and mSIN1 which further increased upon exposition to Pyrogallol for 24 h. **h** MDA-MB-231 and DU 145 cells pre-treated with Ras inhibitors viz. FTI/GGTI (1 μM/38 nM) for 24 h exhibited diminished interaction between mSIN1 and Ras despite Pyrogallol exposition. **i** In an in vitro reconstitution assay wherein constitutive Ras activation was achieved by incubating MDA-MB-231 and DU 145 cell lysates with GTP-γ-S (200 μM) for 12 mins. The co-immunoprecipitation study revealed increased interaction between mSIN1 and active Ras. **j** HA pull-down assay in ectopically HA-Ras expressing MDA-MB-231 and DU 145 cells exhibited increased co-immunoprecipitation of mSIN1 with HA-Ras, following exposition to Pyrogallol. All data are representative of three independent experiments. ***P ≤ 0.001

Next, we set out to explore the mechanism by which superoxide anion may regulate mTORC2 signaling cascade. Recent studies conducted in *Dictyostelium discoideum* suggested the role of Ras in the upstream regulation of TORC2 [14]. Additionally, Heo et al. (2005) reported activation of Ras by superoxide anions via a radical-based mechanism [17]. This led us to probe for possible involvement of Ras during superoxide anion-induced mTORC2 signaling. Results of our immunocytochemical and subcellular fractionation studies revealed that MDA-MB-231 and DU 145 cells stimulated with Pyrogallol exhibited increased redistribution of Ras to the plasma membrane which in turn was an indicator of heightened Ras activation (Fig. 2a, b). Ras activation and its increased localization to membrane-proximal regions with concurrent mTORC2 activation following Pyrogallol stimulus pointed towards an association between superoxide anions, mTORC2 signaling, and Ras. To confirm this, we next set out to study the effect of Ras inhibition on Pyrogallol-induced mTORC2 activation. Ras activation requires a post-translational modification such as farnesylation/geranylation, to dock at the plasma membrane where it is activated by guanine nucleotide exchange factors (GEFs), which catalyze the binding of GTP in place of GDP [28]. We exploited this post-translational modification of Ras for inhibiting Ras activation. MDA-MB-231 and DU 145 cells pre-treated with farnesyltransferase/geranylgeranyltransferase inhibitors (FTI/GGTI) prior to Pyrogallol stimulation exhibited attenuated mTORC2 signaling as evident from diminished p-Ser^2481^mTOR, p-Ser^473^AKT and p-Ser^657^PKC-α levels as compared to cells treated with Pyrogallol alone (Fig. 2c;Additional file 1: Figure S6A). Our results illustrated that pre-treatment of cells with FTI/GGTI prevented the superoxide anion mediated activation of mTORC2.

To better substantiate the critical role of Ras in superoxide anion-mediated activation of mTORC2, we next examined the effect of constitutive activation of Ras employing GTP-γ-S on mTORC2 activation in a cell-free in-vitro reconstitution assay. GTP-γ-S is a non-cell permeable, non-hydrolysable analog of GTP which renders G-proteins constitutively active. We observed that mTORC2 was rapidly phosphorylated at Ser^2481^ as well as AKT/PKC-α at Ser^473^ and Ser^657^ respectively, within 12 min of addition of GTP-γ-S to cell lysate (Fig. 2d;Additional file 1: Figure S6B). Additionally, cells that were made to overexpress Ras ectopically, exhibited potentiated mTORC2 signaling which in turn was more pronounced in Pyrogallol-stimulated Ras overexpressing cells. Simultaneously, we observed elevated levels of pSer^2481^mTOR, pSer^473^AKT and pSer^657^PKC-α in cells overexpressing a constitutively active mutant (G12 V) *RAS* (Fig. 2e;Additional file 1: Figure S6C). Together, these results confirmed the role of Ras in mTORC2 signaling.

The mSIN1 is an integral component of mTORC2 necessary for the assembly of the mTORC2 complex [29]. It contains a phospholipid-binding pleckstrin homology (PH) domain that is postulated to mediate the binding of mTORC2 to membranes [30]. Pertinently, Schroder et al., (2007) reported that mSIN1 also contains a RBD through which it interacts with active Ras [19]. In addition, Yao et al. (2017) identified mSIN1 amino acids 260–460 as a key constituent region of the binding interface with H-Ras [18]. We next asked whether Ras mediates superoxide anion stimulated mTORC2 activation via its interaction with mSIN1 and tested the effect of superoxide stimulus on Ras-mSIN1 interaction. Co-immunoprecipitation studies revealed that superoxide anion stimulus elevated Ras-mSIN1 interaction as compared to control (Fig. 2f). These results were further substantiated through in situ Proximity ligation assay wherein a direct physical interaction was observed between mSIN1 and Ras, which further increased following treatment with Pyrogallol (Fig. 2g). To determine whether Ras inhibition hampered Ras-mSIN1 interaction, we treated MDA-MB-231 and DU 145 cells with FTI/GGTI and found that superoxide anion-induced Ras-mSIN1 interaction was abolished in cells pre-treated with FTI/GGTI as compared to cells treated with Pyrogallol alone (Fig. 2h). Simultaneously, FTI treatment prevented Ras localization to the plasma membrane (Additional file 1: Figure S2). We also observed elevated co-immunoprecipitation of Ras-mSIN1 in lysate incubated with GTP-γ-S in an in-vitro reconstitution assay (Fig. 2i). Finally, we set out to see whether superoxide anion stimulus affects the binding of ectopically expressed Ras with mSIN1, we expressed HA-tagged Ras in MDA-MB-231 and DU 145 cell lines. We observed that superoxide anion stimulation led to enhanced Ras-mSIN1 interaction as evident from the results of co-immunoprecipitation studies (Fig. 2j). Collectively, these results demonstrate that superoxide anions activate mTORC2 in a Ras-dependent manner and Ras-mSIN1 interaction may play an important role in this phenomenon.

### Disruption of Ras-mSIN1 interaction ablates superoxide anion-stimulated mTORC2 activation

**Fig. 3:**
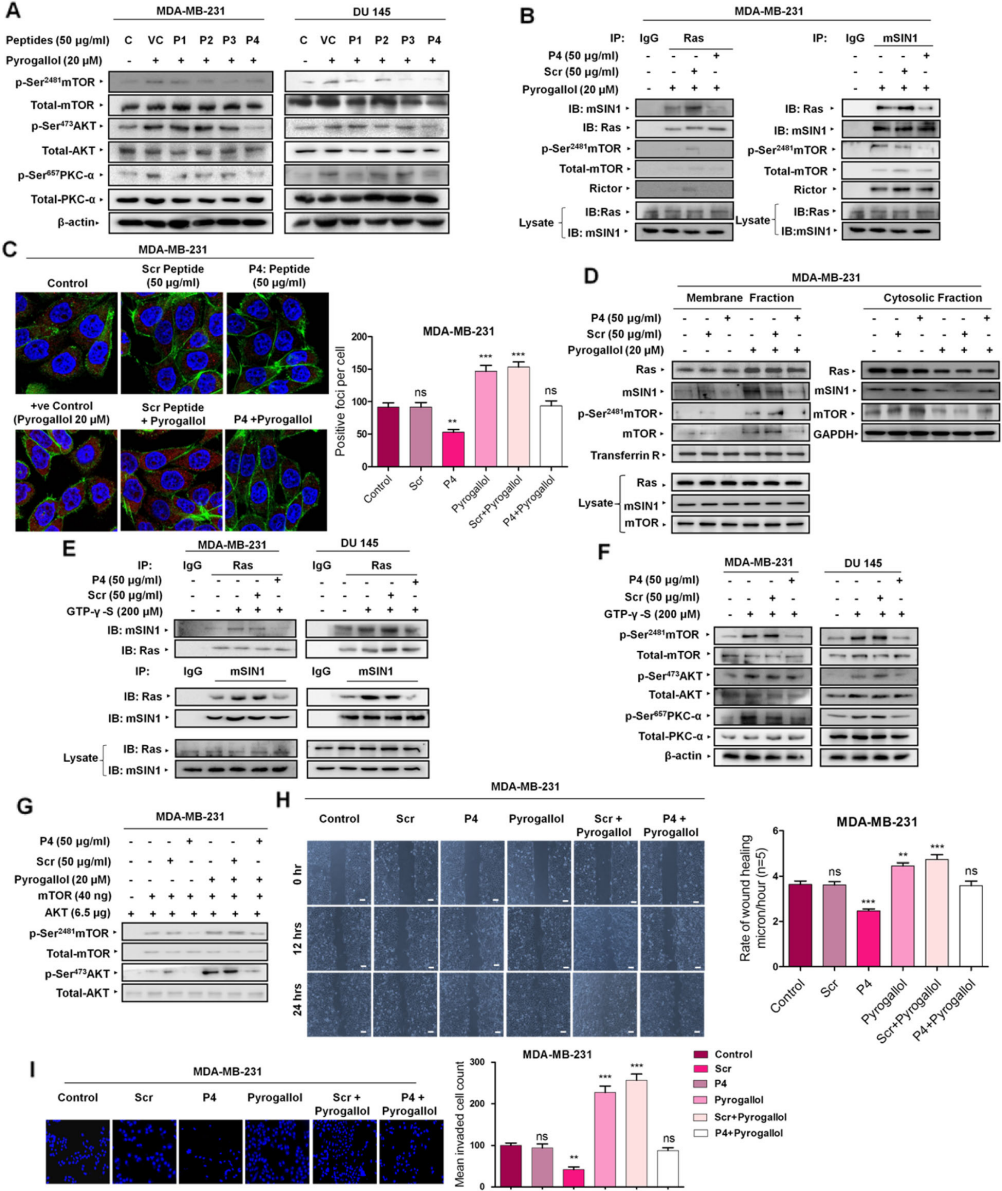
Disruption of Ras-mSIN1 interaction leads to the inhibition of mTORC2 signaling. **a** Pre-treatment (24 h prior to Pyrogallol) of MDA-MB-231 and DU 145 cells with peptides synthesized on the basis of the amino acid sequence of the RBD within mSIN1 revealed diminished mTORC2 signaling compared to that in cells treated with Pyrogallol (20 μM) alone. Maximum inhibition was observed with peptide designated P4. **b** Co-immunoprecipitation studies carried out in MDA-MB-231 cells pre-treated with P4 and Scr peptide for 24 h followed by Pyrogallol stimulation revealed diminished interaction between Ras and mSIN1 in presence of P4 compared to that in Pyrogallol treated or cells treated with scrambled peptide. **c** In situ proximity ligation assay (PLA) revealed that direct physical interaction between Ras and mSIN1 was hampered in cells that were pre-treated with P4. **d** Western blot analysis of cytosolic fraction, membrane fraction and whole cell lysate revealed increased levels of Ras and mSIN1 in membrane fraction with a corresponding decline in the cytosolic fraction following Pyragallol treatment. Pre-treatment with Scrambled peptide did not alter cellular redistribution of either Ras or mSIN1. Pre-treatment with P4 diminished mSIN1 localization to membrane fraction without altering Ras levels in the membrane fraction. The mTOR followed a pattern similar to mSIN1. **e** Constitutive Ras activation with GTP-γ-S treatment for 12 mins in cell lysates of un-treated MDA-MB-231 and DU 145 cells, and ensuing increased interaction of Ras with mSIN1 has hindered cells that were treated with P4 (24 h prior to harvest). **f** Constitutive Ras activation with GTP-γ-S treatment for 12 mins in cell lysates of un-treated MDA-MB-231 and DU 145 cells and ensuing mTORC2 signaling was hindered in cells that were treated with P4 (24 h prior to harvest). **g** The Immunoblot analysis of in vitro kinase assay using mTORC2 immunopurified from MDA-MB-231 cells treated with P4 either alone or in combination with Pyrogallol shows diminished kinase activity as compared to reactions containing mTORC2 immunopurified from Pyrogallol treated or untreated cells. **h** MDA-MB-231 cells treated with P4 (50 μg/ml for 24 h) prior to Pyrogallol exposition (20 μM for the further 12 and 24 h) exhibited diminished cell migration compared to those that were pre-treated with scrambled Peptide. Scale bars: 100 μm. **i** MDA- MB-231 cells treated with P4 (50 μg/ml for 24 h) prior to Pyrogallol exposition (20 μM for the further 24 h) exhibited diminished invasion through Matrigel. Cells were stained with DAPI. *n* = 3 biological replicates per group. All data are representative of three independent experiments. ns (not significant). *P ≤ 0.05, **P ≤ 0.01 and ***P ≤ 0.001

Our previous results demonstrated the involvement of Ras in superoxide anion-mediated potentiation of mTORC2 signaling. We observed that a superoxide anion stimulus led to enhanced Ras-mSIN1 interaction which correlated with potentiated mTORC2 signaling. We next decided to hinder Ras-mSIN1 interaction to evaluate its effect on mTORC2 activation. We evaluated the effect of synthetic peptides of sequences that were identical to the Ras-binding domain RBD of mSIN1. As mSIN1 interacts with Ras through its RBD, we hypothesized that the competitive binding of these peptides might disrupt Ras/mSIN1 interaction. On the basis of Schroder et al (2007) five rationale design-based (Additional file 1: Figure S3) peptides (P1, P2, P3, P4, and P5) of varied amino acid sequences were synthesized (Table 1). P5 was excluded from the study due to non-solubility in any solvent. We initially tested these peptides for any effect on the superoxide anion-stimulated activation of mTORC2. Though all the peptides had mTORC2 inhibitory properties at 50 μg/ml concentration, P4 exhibited maximum inhibitory potential in MDA-MB-231 and DU 145 cell lines (Fig. 3a;Additional file 1: Figure S7A). Based on these results P4 was selected for further detailed investigations. However, prior to embarking on further studies, we set out to ascertain if P4 was capable of gaining intracellular access. To this end, we incubated MDA-MB-231 cells with FITC conjugated P4 for 24 h followed by immunofluorescence analysis which confirmed intracellular accessibility of P4 (Additional file 1: Figure S3A). Next, we decided to evaluate the effect of P4 on Ras-mSIN1 interaction. Our results demonstrated that pre-treatment of MDA-MB-231 cells with P4 lead to diminished Ras-mSIN1 interaction in cells stimulated with Pyrogallol as evident from the co-immunoprecipitation results (Fig. 3b). We next confirmed the results by analyzing the effect of P4 on Ras-mSIN1 interaction by in situ proximity ligation assay. We observed superoxide anion mediated Ras-mSIN1 interaction was hampered in the presence of P4 (Fig. 3c). Localization of mTORC2 and its downstream targets at the plasma membrane is a prerequisite for the successful commencement of mTORC2 activation. Having demonstrated that active Ras preferentially sequesters in membrane-proximal regions and that mSIN1 a key component of mTORC2 physically interacts with Ras, we next hypothesized that active Ras through its interaction with mSIN1 facilitates translocation of already mSIN1 bound mTOR to the plasma membrane. To test this hypothesis, the cells that were pre-treated with P4 (24 h) followed by Pyrogallol treatment (24 h) were subjected to subcellular fractionation to separate cytosolic and membrane fractions. Western blot analysis of total cell lysate, cytosolic fraction and membrane fractions revealed that pyrogallol treatment resulted in increased levels of Ras and mSIN1 in the plasma membrane fraction with a corresponding decline in the cytosolic fraction (Fig. 3d). While scrambled peptide did not alter pyrogallol mediated sequestration of either of these to membrane fraction, the P4 markedly diminished mSIN1 levels in membrane fraction despite similar Ras levels in the membrane fraction. Corresponding to this, the cytosolic levels of mSIN1 were much elevated in cells pre-treated with P4 despite lower levels of Ras. Interestingly the p-Ser ^2481^mTOR levels were also markedly declined in presence of P4 and so were pan mTOR levels. Thus mTOR also followed a pattern similar to mSIN1 indicating that mTOR while being bound to mSIN1 translocated to membrane-proximal regions when mSIN1 physically interacted with active Ras, impeding this interaction by way of P4 treatment not only hindered mTOR from localizing to membrane proximal regions but also hampered over all p-Ser^2481^ mTOR levels in membrane fraction. As demonstrated in previous results, the addition of GTP-γ-S to unstimulated MDA-MB-231 and DU 145 cells resulted in enhanced co-immunoprecipitation of Ras with mSIN1 which in turn culminated into enhanced mTORC2 activation. Here, we evaluated that the simultaneous addition of P4 along with GTP-γ-S lowered the amount of Ras-mSIN1-precipitated immune complexes as compared to GTP-γ-S treated lysates (Fig. 3e). Furthermore, the simultaneous addition of P4 negatively modulated mTORC2 activation status even in the presence of GTP-γ-S driven constitutive Ras activation (Fig. 3f;Additional file 1: Figure S7B). Next, we decided to evaluate the inhibitory effect of P4 directly on mTORC2 kinase activity in isolation. To this effect, the in vitro reconstitution assay was carried out. The mTORC2 immunopurified (employing Rictor pull down) from Pyrogallol and/or P4-treated cells, was incubated with immunopurified AKT. Endpoint mTORC2 kinase activity as assessed by Western blot analysis for detecting level of p-Ser^473^ levels revealed decreased in vitro phosphorylation of AKT at Ser^473^ in reaction mixture containing mTORC2 purified from cells that were treated with P4 either alone or in combination with Pyrogallol as compared to that in reaction mixture containing mTORC2 immunopurified from Pyrogallol treated or un-treated MDA-MB-231 cells (Fig. 3g).

**Table 1:** Rationale based peptides

Peptides	Amino Acid Sequences
Peptide-1 (P1)	SKESLFVRINAAHGFS
Peptide-2 (P2)	SLIQVDNTKVTMKEI
Peptide-3 (P3)	LLKAVKRRKGSQK
Peptide-4 (P4)	VSGPQYRLEKQSEPNV
Peptide-5 (P5)	AVDLDSTLESQSAWEFCLVR
Scrambled Peptide	SVYGNEPQRQLVSEPK

As we finally demonstrated that Ras-mSIN1 interaction is important for Ras-mediated potentiation of mTORC2 signaling, we next set out to evaluate the effect of P4 on cancer cell migration and invasion potential, one of the important functional outcomes of activated mTORC2 signaling. A multitude of studies suggests that activation of mTORC2 signaling induces cancer cell migration and invasion [31–33]. To assess the effect of P4 on migration, we performed cell migration or scratch assay in the presence and absence of Pyrogallol. MDA-MB-231 pre-treated with P4 exhibited significantly diminished cell migration as such, as well as in presence of Pyrogallol, as indicated by the extent of gap closure by the end of the treatment period of 24 h (Fig. 3h). Similar results were found with P4 in the cell invasion potential of MDA-MB-231 cells (Fig. 3i). Thus, our results reveal that disrupting Ras-mSIN1 interaction by treating cells with P4 inhibits mTORC2 functions per se as well as despite Pyrogallol stimulation as evident from diminished cell migration and invasion potential.

**Fig. 4:**
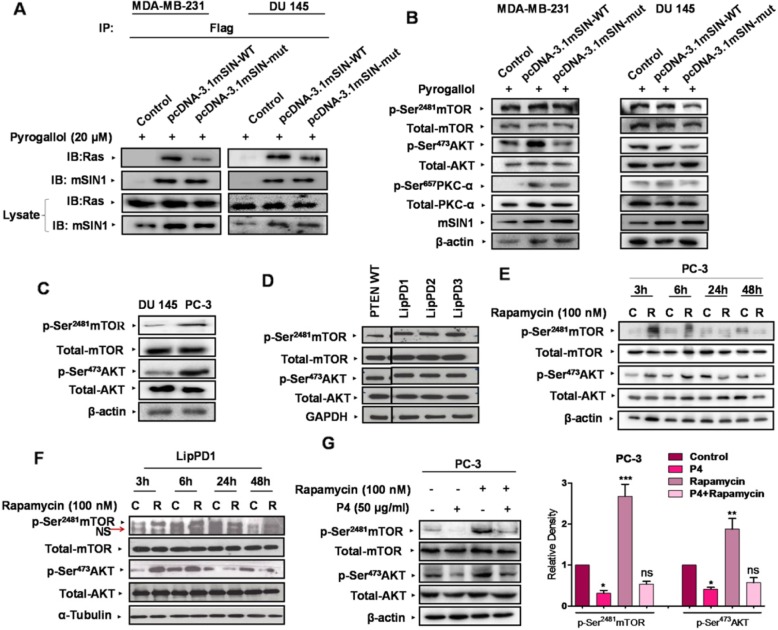
**a** MDA-MB-231 and DU 145 cells were transfected with control vector, wild-type FLAG-mSIN1, and mutant FLAG-mSIN1-containing expression vectors followed by Pyrogallol stimulation for 24 h. Compared to MDA-MB-231 and DU 145 cells overexpressing Flag-tagged, wild-type mSIN1, the ones expressing Flag-tagged, mutant mSIN1 (Tyr-323 and Leu-325 replaced with Ala) exhibited diminished Ras-mSIN1 interaction. **b** Compared to cells overexpressing Flag-tagged- wild-type mSIN1, the ones expressing Flag-tagged-mutant mSIN1 (Tyr-323 and Leu-325 replaced with Ala) exhibited diminished mTORC2 signaling. **c** PTEN-deficient cell line PC-3 exhibited elevated activation of mTORC2 compared to DU 145 cells as shown by Immunoblot analysis. **d** Western blot analysis of lysates from PTEN-deficient LipPD1, LipPD2, LipPD3 lipoma cells exhibited diminished activation of mTORC2 signaling cascade as compared to PTEN wildtype-preadipocytes. **e** Western blot analysis of lysates from PC-3 cells treated with Rapamycin for various time points shows heightened mTORC2 signaling at 3 h and gradually decreases. **f** LipPD1 cells treated with Rapamycin at various time points exhibited heightened mTORC2 activation with most prominent effects at 3 h post-treatment. **g** Immunoblot analysis of cell lysates of PC-3 cells, pre-treated with P4 for 24 h followed by rapamycin for 3 h as indicated. Pre-treatment with P4 show decreased activation of mTORC2, alone and in combination with Rapamycin. All data are representative of three independent experiments. ns, not significant. *P ≤ 0.05, **P ≤ 0.01 and ***P ≤ 0.001

Our detailed analysis of the P4 amino acid sequences from RBD leads us to pinpoint two of the amino acid residues, Tyr-323(Y^323^) and Leu-325(L^325^), within the region corresponding to P4 that were conserved across the species (Additional file 1: Figure S3). Among these two amino acids, tyrosine has the third-highest conservation propensity on binding sites offering a hydrophobic surface for binding to the other interacting protein [34]. Based on these observations and previous reports it was apparent that this region within RBD is involved in the Ras-mSIN1 interaction. To confirm that this site mediates the interaction of mSIN1 with Ras and subsequently confirm its effect on mTORC2 signaling, we sought to mutate these conserved amino acids with alanine substitutions for our study (Additional file 1: Figure S4). We created Flag-tagged wild-type and mutant mSIN1 constructs, denoted in this study as WT-Flag-pcDNA-3.1 and Mutant-Flag-pcDNA-3. We ectopically expressed these in MDA-MB-231 and DU 145 and did a Flag-immunoprecipitation assay. Interestingly, our results revealed decreased Ras-mSIN1 interaction in cells transfected with mutant mSIN1 as depicted in immuno-blot images (Fig. 4a). Our result also confirmed that cell lysate from mutant mSIN1-transfected cells exhibited diminished mTORC2 activation (Fig. 4b;Additional file 1: Figure S8A).

One of the important pathophysiological conditions associated with the hyperactive PI3K/AKT/mTOR axis is deficient PTEN function such as is observed in PTEN-deficient cancer cells or lipoma cell cultures derived from PTEN hamartoma tumor syndrome (PHTS) patients [20]. In order to see whether Ras-mSIN1 interaction plays a role in the activation of mTORC2 via other stimuli, such as PTEN deficiency and rapamycin treatment, we next examined the effect of P4 in the PTEN-deficient cell line PC-3 and PTEN-haploinsufficient lipoma cells LipPD1, LipPD2, and LipPD3 [35]. Since PTEN deficiency accounts for elevated PIP3 levels and PIP3s directly activate mTORC2 kinase function [36], it only appeared plausible that PTEN-deficient cells may exhibit heightened mTORC2 signaling. In agreement with this, the PTEN-deficient prostate cancer PC-3 cells exhibited hyperactive mTORC2 signaling as compared to PTEN intact DU 145 cells (Fig. 4c;Additional file 1: Figure S8B). Similarly, the lipoma cell cultures LipPD1, LipPD2 and LipPD3 derived from patients with PHTS exhibited heightened mTORC2 signaling (Fig. 4d) as compared to PTEN wildtype pre-adipocytes. Treatment of PC-3 cells with rapamycin for different time intervals further potentiated mTORC2 signaling and AKT activation with the most prominent effects at 3 h post-treatment (Fig. 4e). Similarly, the PHTS patient-derived LipPD1 cells also exhibited heightened mTORC2 signaling with the most prominent effects at 3 h post-treatment (Fig. 4f). This, in turn, could be the underlying basis for rapamycin resistance observed in such cellular systems with diminished or absent PTEN activity [20]. Interestingly, treatment with P4 not only diminished the basal level of mTORC2 signaling in PTEN-deficient PC-3 cells, but pre-treatment with P4 also circumvented the rapamycin-induced AKT and mTOR phosphorylation (Fig. 4g). Thus, our results indicated that Ras-mSIN1 interaction could be a critical regulatory switch governing the mTORC2 signaling cascade in PTEN-deficient cells. Collectively, these results emphasize the significance of the physical interaction between Ras and mSIN1 during the functional regulation of mTORC2.

## Discussion

The mTOR complexes serve as important regulators of cellular homeostasis and are clinically important drug targets. Although much has been revealed about the regulation and functioning of mTORC1 with the discovery of rapamycin, limited knowledge about the regulation of mTORC2 has stalled the progress to develop mTORC2-specific inhibitors and finally the development of an efficient mTOR-specific therapeutic strategy. The current study sheds light on potential upstream regulatory mechanisms governing the mTORC2 signaling pathway. Here, we report a key regulatory role of Ras in mTORC2 signaling wherein Ras via its interaction with mSIN1, potentiates mTORC2 cascade in cancer cells.

While characterizing the pathway through which superoxide anions affected mTORC2 activation, it was observed that superoxide anion dependent mTORC2 potentiation correlated with enhanced localization of Ras towards the plasma membrane and enhanced binding of mSIN1 with Ras. As reported earlier mSIN1 binds to active GTP-bound Ras through its Ras-binding domain RBD indicating the involvement of Ras in the postulated pathway. For both the pathways i.e. Ras and mTORC2, one of the critical events for successful activation and further relay of signals is their correct subcellular localization. The association of Ras with the plasma membrane is mediated by various post-translational modifications. Inhibition of the post-translational modification and consequently its plasma membrane localization leads to the loss of biological activity of Ras [37, 38]. A possible role of Ras in the superoxide anion-stimulated activation of mTORC2 came from the reports that (i) Ras is a superoxide sensitive protein and is activated by superoxide anions via a radical-based mechanism [17] and (ii) localization of mTORC2 and its downstream targets at the plasma membrane is a prerequisite for successful commencement of mTORC2 activation [39]. In support of this, we first demonstrated that attenuating Ras activation by inhibiting post-translational farnesylation/ geranylation resulted in diminished Ras-mSIN1 interaction which in turn culminated in impeded mTORC2 signaling despite Pyrogallol-induced superoxide anion stimulus. Secondly, the constitutive activation of Ras with GTP-γ-S in an in-vitro assay enhanced both Ras-mSIN1 interaction and mTORC2 activation. Thirdly, the ectopic expression of Ras potentiated mTORC2 activity which was further enhanced by superoxide stimulation. Interestingly, cells expressing constitutively active Ras (G12 V) exhibited maximum activation of mTORC2 thereby pointing towards the possibility of active Ras also being a standalone potentiator of mTORC2 cascade. This finding holds special significance with respect to the Ras driven cancers wherein oncogenic mutations impart a constitutively active phenotype to Ras proteins.

**Fig. 5:**
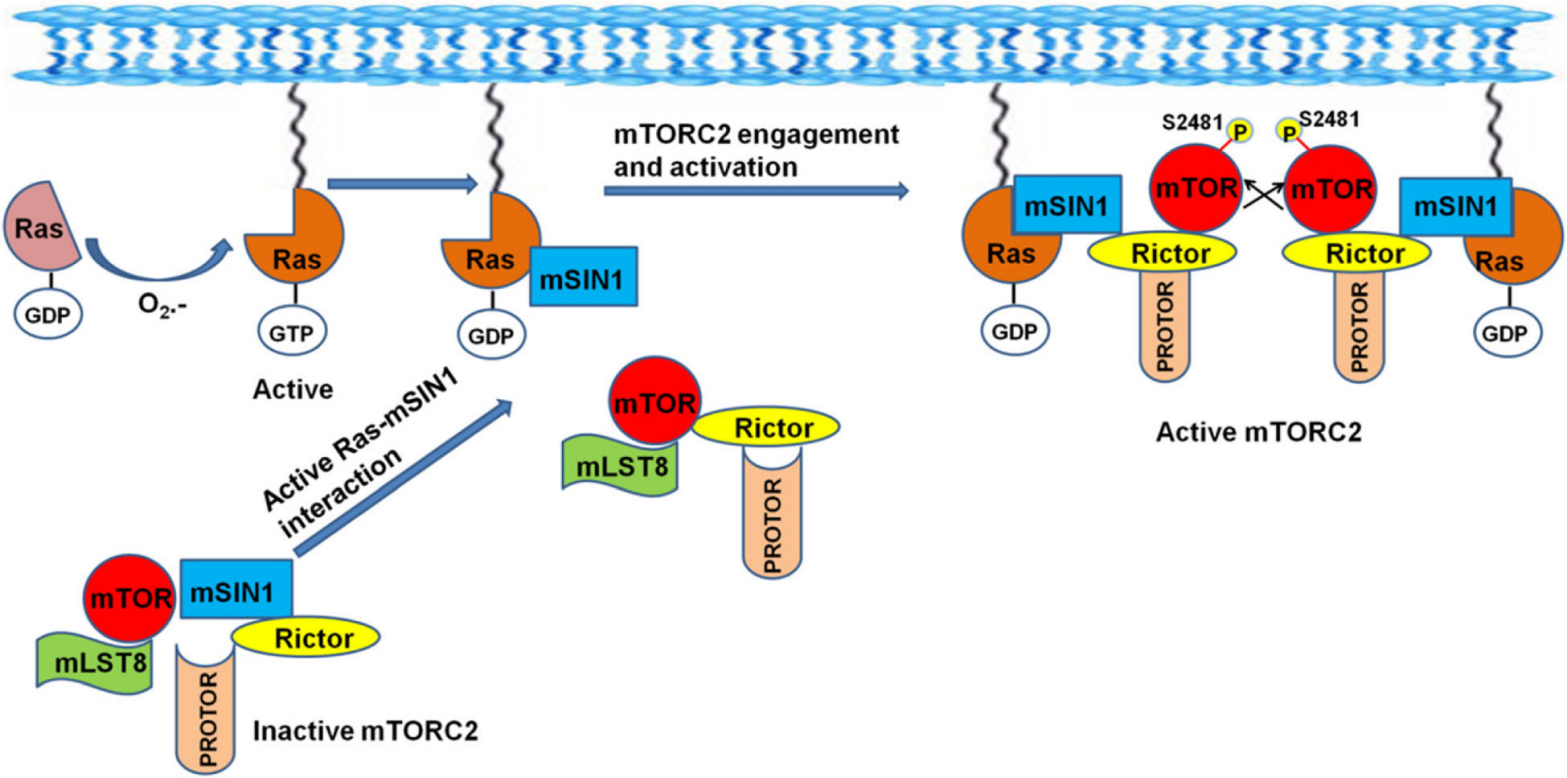
Schematic representation of Ras activation by superoxide anion and its localization at the plasma membrane leading to mTORC2 activation

We next questioned whether the Ras-mSIN1 binding is a critical event in the Ras-mediated activation of mTORC2. We employed synthetic peptide-based as well as genetic approaches to disrupt the Ras-mSIN1 interaction and evaluated its effect on mTORC2 activation. Our first evidence to confirm the role of Ras-mSIN1 interaction in superoxide anion-stimulated mTORC2 activation emerged when synthetic peptides (corresponding to sequences from the Ras binding domain of mSIN1) inhibited mTORC2 activation. Our further studies with P4, which exhibited maximum mTORC2 inhibition, demonstrated that inhibition of Ras-mSIN1 interaction not only impeded translocation of mSIN1 to membrane fraction, but it also impeded mTOR from sequestering into membrane-proximal regions. These results suggested that active Ras through its interaction with mSIN1 facilitated translocation of mTOR also to the plasma membrane. It is plausible that in doing so, Ras not only places mSIN1/mTOR at a subcellular locale that is conducive to autophosphorylation of mTOR at Ser^2481^, an event that commits mTOR to nucleate into mTORC2, but also places mSIN1-mTOR heterodimer in close proximity to downstream effector molecules such as AKT and PKC-α that are known to dock at plasma membrane through their PH domain during their activation. Thus, our study revealed the crucial role of Ras in translocating mSIN1/mTOR heterodimer to the plasma membrane and thereby facilitating the activation and commencement of mTORC2 cascade (Fig. 5).

Rapamycin is known to inhibit the only mTORC1, with no direct inhibitory effect on mTORC2. In fact, the inhibitory effect of Rapamycin on mTORC2 is observable only at later time points viz. 24 to 72 h [27, 40] which is an indirect consequence of unavailability of free mTOR to nucleate into mTORC2 due to it being engaged with Rapamycin during prolonged treatment. In agreement with this, we also observed mTORC2 inhibition 24 h after Rapamycin treatment. However, at earlier time points i.e. 1 to 3 h, Rapamycin treatment resulted in heightened mTORC2 signaling in agreement with O’Riley et al. [12]. Considering the key role of heightened mTORC2 signaling in developing Rapamycin resistance, the ability of P4 to inhibit mTORC2 under these conditions has significant clinical implications in terms of countering the treatment resistance. Additionally, successful inhibition of mTORC2 in PTEN deficient cells (where PDKs are already in the hyperactivated state) via the experimental strategy of targeting Ras-mSIN1 interaction is another significant finding having the potential for application in managing tumors emanating from deficient PTEN function.

By establishing Ras as an upstream regulatory switch governing mTORC2 cascade, our study paves the way to possibilities for specific inhibition of mTORC2. Not only does our study establishes Ras as an upstream regulatory element to mTORC2 cascade, but it also establishes mTORC2 as a downstream effector of Ras pathway. About one-third of all human cancers exhibit oncogenic mutations in *RAS* isoforms (*KRAS, NRAS, and HRAS)* making Ras an important anti-cancer target [41]. The emergence of mTORC2/Ras crosstalk via mSIN1 additionally opens a new avenue wherein specific targeting of mTORC2 could also serve as a potential strategy for the treatment of oncogenic Ras driven cancers as well.

## Conclusions

In summary, we have identified superoxide anion as an upstream activator of mTORC2. We have also established the role of Ras in the upstream regulation of mTORC2 signaling with the discovery that Ras-mSIN1 interaction is a key event during the superoxide anion mediated activation of mTORC2. Employing cell migration and cell invasion index as a measure of cellular function, we established that disruption of Ras-mSIN1 interaction impedes mTORC2 dependent cellular functions. These findings could serve as the basis for the design and development of selective mTORC2 inhibitors, which in turn could be potentially used as an effective combinatorial anti-cancer regimen.

## Supplementary information

**Additional file 1 **: **Figure S1.** Superoxide anion generation in Pyrogallol treated cells**. (A**) MDA-MB-231 cells were treated with Pyrogallol (10, 20, 50 and 100 μM) for 24 h followed by 30 mins incubation with 10 μM DHE and analyzed for superoxide anion detection using a fluorescence microscope (above). Phase-contrast images of cells after indicated concentration of Pyrogallol treatment (below). Scale bars, 50 μm. **(B)** MDA-MB-231 cells were treated with 20 μM Pyrogallol for time point as indicated in the figure, and Western blotting was done for mTORC2 specific markers. All data are representative of three independent experiments. **Figure S2.** Pyrogallol prevents Ras localization to the plasma membrane. MDA-MB-231 cells pre-treated with FTI (Lonafarnib 1 μM) for 4 h followed by stimulation with Pyrogallol (20 μM) for another 24 h. Cells were analyzed for Ras localization by immunofluorescence microscopy. Data are representative of three independent experiments. **Figure S3.** Evaluation of sequences of peptides. Evaluation of sequences of synthetic peptides identical to the Ras-binding domain (RBD) of mSIN1. **Figure S4.** Peptide penetration and mutation analysis. **(A)** Immunofluorescence images of MDA-MB-231 cells treated with FITC-conjugated P4 (50 μg/ml) for 24 h. **(B)** RBD sequences of wild-type and mutant. Two of the amino acid residues Tyr-323(Y^323^) and Leu-325(L^325^) within the region corresponding to P4 in the wild-type RBD, were conserved across the species. The conserved amino acids tyrosine and leucine were mutated with alanine substitutions. **Figure S5.** Quantification of data of Fig. 1b Densitometric quantification of protein phosphorylation of mTORC2 specific markers by Western blot data (represented in Fig. 1b). **P ≤ 0.01, ***P ≤ 0.001. **Figure S6.** Quantification of data of Fig. 2c, d, and e. **(A)** Densitometric quantification of protein phosphorylation of mTORC2 specific markers by Western blot data (represented in Fig. 2c). **(B)** Densitometric quantification of protein phosphorylation of mTORC2 specific markers by Western blot data (represented in Fig. 2d). **(C)** Densitometric quantification of protein phosphorylation of mTORC2 specific markers by Western blot data (represented in Fig. 2e). Pyr (Pyrogallol) ns (not significant). *P ≤ 0.05, **P ≤ 0.01, ***P ≤ 0.001. **Figure S7.** Quantification of data of Fig. 3a and f. **(A)** Densitometric quantification of protein phosphorylation of mTORC2 specific markers by Western blot data represented in Fig. 3a. **(B)** Densitometric quantification of Western blot data represented in Fig. 3f. VC (Vehicle control), Pyr (Pyrogallol), and ns, not significant. *P ≤ 0.05, **P ≤ 0.01, ***P ≤ 0.001. **Figure S8.** Quantification of data of Fig. 4b and c. Densitometric quantification of protein phosphorylation of mTORC2 specific markers by Western blot data (represented in Fig. 4b). ns, not significant. *P ≤ 0.05, **P ≤ 0.01, ***P ≤ 0.001.

## Data Availability

The datasets used and/or analyzed during the current study are available from the corresponding author on reasonable request.

## Abbreviations

DEPTOR: DEP domain containing mTOR interacting protein
DTT: Dithiothreitol
ER: Estrogen receptor
FTI: Farnesyl transferase inhibitor
GGTI: Geranylgeranyl transferase inhibitor
GTP: Guanosine triphosphate
mLST8: Mammalian lethal with SEC13 protein 8
mSIN1: Mammalian stress-activated protein kinase (SAPK) interacting protein 1
mTOR: Mechanistic (or mammalian) target of rapamycin
mTORC1: Mechanistic (or mammalian) target of rapamycin complex 1
mTORC2: Mechanistic (or mammalian) target of rapamycin complex 2
NMR: Nuclear Magnetic Resonance
PHTS: PTEN hamartoma tumor syndrome
PI3K: Phosphatidylinositol 3-kinase
PIKK: Phosphatidylinositol 3-kinase-related kinase
PKC-α: Protein kinase C alpha
PRAS40: Proline-rich AKT substrate 40
PROTOR: Protein observed with Rictor
PTEN: Phosphatase and tensin homolog
RAF: Rapidly accelerated fibrosarcoma
Raptor: Regulatory-associated protein of mTOR
RBD: Ras binding domain
Rheb: Ras homolog enriched in brain
Rictor: Rapamycin insensitive companion of mTOR
ROS: reactive oxygen species
RP-HPLC: Reversed phase high-performance liquid chromatography
SAPK: Stress activated protein kinase
SOD2: Superoxide dismutase 2
SPPS: Solid phase peptide synthesis
