# Supplementary material for: Direct physical interaction of active Ras with mSIN1 regulates mTORC2 signaling
Source: BMC Cancer. 2019 Dec 19;19:1236. doi: 10.1186/s12885-019-6422-6 (PMC6921532; doi:10.1186/s12885-019-6422-6)
Supplement: Supplementary file 1 — Additional file 1 : Figure S1. Superoxide anion generation in Pyrogallol treated cells. (A) MDA-MB-231 cells were treated with Pyrogallol (10, 20, 50 and 100 μM) for 24 h followed by 30 mins incubation with 10 μM DHE and analyzed for superoxide anion detection using a fluorescence microscope (above). Phase-contrast images of cells after indicated concentration of Pyrogallol treatment (below). Scale bars, 50 μm. (B) MDA-MB-231 cells were treated with 20 μM Pyrogallol for time point as indicated in the figure, and Western blotting was done for mTORC2 specific markers. All data are representative of three independent experiments. Figure S2. Pyrogallol prevents Ras localization to the plasma membrane. MDA-MB-231 cells pre-treated with FTI (Lonafarnib 1 μM) for 4 h followed by stimulation with Pyrogallol (20 μM) for another 24 h. Cells were analyzed for Ras localization by immunofluorescence microscopy. Data are representative of three independent experiments. Figure S3. Evaluation of sequences of peptides. Evaluation of sequences of synthetic peptides identical to the Ras-binding domain (RBD) of mSIN1. Figure S4. Peptide penetration and mutation analysis. (A) Immunofluorescence images of MDA-MB-231 cells treated with FITC-conjugated P4 (50 μg/ml) for 24 h. (B) RBD sequences of wild-type and mutant. Two of the amino acid residues Tyr-323(Y323) and Leu-325(L325) within the region corresponding to P4 in the wild-type RBD, were conserved across the species. The conserved amino acids tyrosine and leucine were mutated with alanine substitutions. Figure S5. Quantification of data of Fig. 1b Densitometric quantification of protein phosphorylation of mTORC2 specific markers by Western blot data (represented in Fig. 1b). **P ≤ 0.01, ***P ≤ 0.001. Figure S6. Quantification of data of Fig. 2c, d, and e. (A) Densitometric quantification of protein phosphorylation of mTORC2 specific markers by Western blot data (represented in Fig. 2c). (B) Densitometric quantification of protein pho [file 12885_2019_6422_MOESM1_ESM.docx]

**Direct physical interaction of active Ras with mSIN1 regulates mTORC2 signaling**

**Authors:** Mehraj-U-Din Lone^1,†^, Javed Miyan^1,2†^ Mohammad Asif^1,^ , Showkat A. Malik^1^, Parul Dubey^3^, Varsha Singh^1^, Kavita Singh^4^, Kalyan Mitra^2,4^, Deepali Pandey^5^, Wahajul Haq^5^, Himanshi ^1^, Prince Kumar Singh^1^, Wieland Kiess^6^, Franziska Kaessner^6^, Antje Garten^6,7^, Smrati Bhadauria^1,2,*^

**Supplementary Materials:**

**Materials and Methods**

**Measurement of superoxide anion level**

Intracellular superoxide anions were measured using DHE (Calbiochem, USA) probe. Cells were grown with normal FBS containing media then switched to CS-FBS containing. After 24 hrs, 10 µM DHE was given in each well for 30 mins. Images were taken by using a Leica DCF450C inverted fluorescence microscope attached with a digital camera.

**Figures:**

**Fig. S1. Superoxide anion generation in Pyrogallol treated cells. (A**) MDA-MB-231 cells were treated with Pyrogallol (10, 20, 50 and 100 µM) for 24 hrs followed by 30 mins incubation with 10 μM DHE and analyzed for superoxide anion detection using a fluorescence microscope (above). Phase contrast images of cells after indicated concentration of Pyrogallol (below). Scale bars, 50 µm. **(B)** MDA-MB-231 cells were treated with 20 µM Pyrogallol for time point as indicated in the figure, and Western blotting was done for mTORC2 specific markers. All data are representative of three independent experiments.

**Fig. S2. Pyrogallol prevents Ras localization to the plasma membrane.** MDA-MB-231 cells pre-treated with FTI (Lonafarnib 1 μM) for 4 hrs followed by stimulation with Pyrogallol (20 μM) for another 24 hrs. Cells were analyzed for Ras localization by immunofluorescence microscopy. Data are representative of three independent experiments.

**Fig. S3. Evaluation of sequences of peptides.** Evaluation of sequences of synthetic peptides identical to the Ras-binding domain (RBD) of mSIN1.

**Fig. S4. Peptide penetration and mutation analysis. (A)** Immunofluorescence images of MDA-MB-231 cells treated with FITC-conjugated P4 (50 μg/ml) for 24 hrs. **(B)** RBD sequences of wild-type and mutant. Two of the amino acid residues Tyr-323(Y^323^) and Leu-325(L^325^) within the region corresponding to P4 in the wild-type RBD, were conserved across the species. The conserved amino acids tyrosine and leucine were mutated with alanine substitutions.

**Fig. S5. Quantification of data of fig. 1B** Densitometric quantification of protein phosphorylation of mTORC2 specific markers by Western blot data (represented in Fig. 1B). ***P*≤ 0.01, ****P*≤ 0.001.

**Fig. S6. Quantification of data of fig. 2C, 2D, and 2E. (A)** Densitometric quantification of protein phosphorylation of mTORC2 specific markers by Western blot data (represented in Fig. 2C). **(B)** Densitometric quantification of protein phosphorylation of mTORC2 specific markers by Western blot data (represented in Fig. 2D). **(C)** Densitometric quantification of protein phosphorylation of mTORC2 specific markers by Western blot data (represented in Fig. 2E). Pyr (Pyrogallol) ns (not significant). **P*≤ 0.05, ***P*≤ 0.01, ****P*≤ 0.001.

**Fig. S7. Quantification of data of fig. 3A and 3F. (A)** Densitometric quantification of protein phosphorylation of mTORC2 specific markers by Western blot data represented in Fig. 3A. **(B)** Densitometric quantification of Western blot data represented in Fig. 3F. VC (Vehicle control), Pyr (Pyrogallol), and ns, not significant. **P*≤ 0.05, ***P*≤ 0.01, ****P*≤ 0.001.

**Fig. S8. Quantification of data of fig. 4B and 4C.** Densitometric quantification of protein phosphorylation of mTORC2 specific markers by Western blot data (represented in Fig. 4B). ns, not significant. **P*≤ 0.05, ***P*≤ 0.01, ****P*≤ 0.001.
